# Hydrogen Peroxide Affects Growth of *S. aureus* Through Downregulation of Genes Involved in Pyrimidine Biosynthesis

**DOI:** 10.3389/fimmu.2021.673985

**Published:** 2021-09-07

**Authors:** Hélène Buvelot, Myriam Roth, Vincent Jaquet, Andrey Lozkhin, Adriana Renzoni, Eve-Julie Bonetti, Nadia Gaia, Floriane Laumay, Michéle Mollin, Marie-José. Stasia, Jacques Schrenzel, Patrice François, Karl-Heinz Krause

**Affiliations:** ^1^Department of Pathology and Immunology, Faculty of Medicine, University of Geneva, Geneva, Switzerland; ^2^Service of Infectious Diseases, Department of Medicine, Geneva University Hospitals, Geneva, Switzerland; ^3^Chronic Granulomatous Disease Diagnosis and Research Centre (CDiReC), Pôle Biologie, Centre Hospitaliser Universitaire (CHU) de Grenoble, Grenoble, France; ^4^Université Grenoble Alpes, Comissariat à l'energie atomique (CEA), Centre National de la Recherche Scientifique (CNRS) and Institut de Biologie Structurale (IBS), Grenoble, France; ^5^Genomic Research Laboratory, Department of Medicine, Geneva University Hospitals and Faculty of Medicine, University of Geneva, Geneva, Switzerland

**Keywords:** *S. aureus*, hydrogen peroxide, pyrimidine metabolism, neutrophils, *carA*

## Abstract

Reactive oxygen species (ROS) play a crucial role in the cellular defense against *S. aureus*, as evidenced by the importance of this pathogen in patients lacking the ROS-generating phagocyte NADPH oxidase NOX2. ROS concentrations required to kill *S. aureus in vitro* are much higher than those found in the phagosome. We therefore hypothesized that sublethal ROS concentrations may play a role in *S. aureus* gene dysregulation and investigated the *in vitro* transcriptomic response of *S. aureus* to sublethal concentrations of hydrogen peroxide (H_2_O_2_). A striking observation of these experiments was a coordinated and massive downregulation of genes involved in pyrimidine metabolism. Using transposon insertion mutants, we demonstrated that deletion of *carA*, a gene involved in pyrimidine synthesis, led to a significant growth defect and to an increased sensitivity of *S. aureus* to added H_2_O_2_. The phenotype of the *carA* mutant could be reversed through supplementation with the pyrimidine precursor uracil, or with a multicopy vector encoding *carA*. As opposed to the impact of ROS on extracellular survival, *carA* deletion did not affect the intracellular survival in neutrophils. Our results raise the possibility that ROS-dependent downregulation of pyrimidine metabolism might be a survival strategy of *S. aureus*, allowing colonization through intracellular survival, while decreasing the risk of killing the host through dampened extracellular growth.

## Introduction

*Staphylococcus aureus* is a Gram-positive, round-shaped facultative anaerobic bacterium, discovered in 1881 by Alexander Ogston, a Scottish surgeon. *S. aureus* colonizes approximately 30% of the human population (1) and its main site of colonization is the nasal cavity (2, 3). While *S. aureus* is generally commensal, it causes a broad spectrum of severe infections (4). *S. aureus* is able to adapt to the environment (5) and, in the era of antibiotics, it has rapidly developed or acquired antimicrobial resistances. Development of new anti-staphylococcal treatments is now considered a high priority by the WHO (6).

Reactive oxygen species (ROS) generated by phagocytes are key players in the defense against *S. aureus*. This concept is largely based on the phenotype of chronic granulomatous disease (CGD), a genetic disorder caused by loss of function mutations in the ROS-generating phagocyte NADPH oxidase NOX2. CGD patients often suffer from severe, recurrent and/or persistent infections with *S. aureus* (7). Thus, in a simplistic model, NOX2-derived ROS are killing *S. aureus* by targeting DNA, proteins and lipids (8). However, *S. aureus* possesses a complex antioxidant defense system, including two superoxide dismutases and one catalase (9) and is quite resistant to microbicidal concentrations of ROS. The relationship between ROS and *S. aureus* might be more complex than a direct lethal effect on *S. aureus* and we hypothesized a more subtle mechanism that could affect the bacterial fitness.

Among the different ROS generated by NOX2, hydrogen peroxide (H_2_O_2_) may have a predominant role. High concentration of this non-radical oxidant can damage cells and tissues. However, based on available research, it is unlikely that these very high concentrations (>50 mM) can be reached within the phagosome as phagosomal H_2_O_2_ concentrations are in the micromolar range (10). However, there is increasing evidence that there are H_2_O_2_ gradients and therefore the local H_2_O_2_ concentration in close proximity to the NADPH oxidase might be higher and reach the low millimolar range (11). H_2_O_2_ is now increasingly recognized as an intra- and intercellular signaling messenger (12). H_2_O_2_ can impact cell phenotype through a variety of mechanisms, including regulation of gene expression (13). H_2_O_2_ reversibly oxidizes specific cysteine residues of key protein targets to regulate their function in eukaryotic cells (14). A similar effect of H_2_O_2_ on bacterial signaling was observed (15). For instance, several redox-sensitive transcriptional regulators exist in bacteria (16, 17) and these regulators usually control the expression of genes involved in defense against oxidative stress (18, 19). However, alteration of bacterial signaling by H_2_O_2_ can also be used by host cells as a defense mechanism. Intestinal cells produce low H_2_O_2_ concentrations that interferes with bacterial signaling and weakens the fitness of potential intestinal pathogens (20).

In this study, we addressed a significant open question in the field: concentrations of ROS that can be achieved *in vivo* are sublethal for S. aureus, yet NOX2-derived ROS play a crucial role in the host defense against this microorganism. We found a major impact of sublethal ROS concentrations on gene expression in *S. aureus*. The importance of understanding the interaction of ROS with this important pathogen is severalfold: first, it should provide at least a part of the answer to the oldest question in CGD research, namely why do patients that cannot generate ROS have such difficulties to defend themselves against *S. aureus*; second, a better knowledge of the effect of H_2_O_2_ on *S. aureus* transcriptome might provide new therapeutic targets within *S. aureus*, which is now considered as a high priority pathogen for the development of new treatments by the WHO.

## Materials and Methods

### Bacterial Strains, Culture Medium and Growth Analysis

All bacterial strains used in this study are summarized in **Table 1**. All strains were cultured at 37°C in tryptic soy broth (TSB) or on tryptic soy agar (TSA). When antimicrobial agents were indicated, they were added to the following concentrations: erythromycin (Sigma-Aldrich) 5 µg ml^-1^ and chloramphenicol (AppliChem) 10 µg ml^-1^. For growth restoration of the *carA* mutant strain, the medium was supplemented with uracil 2.5 mM (Sigma-Aldrich).

**Table 1:** Bacterial strains and plasmid.

Strain or plasmid	Relevant genotype or characteristic(s)	Source/reference
*S. aureus* strains		
JE2	*S. aureus* USA300 LAC plasmids-cured	NTML
RN4220	Restriction-defective strain which accepts foreign DNA	(21)
NE1526	JE2-derived strain with a transposon in *carA* gene	NTML
NE1301	JE2-derived strain with a transposon in *pyrB* gene	NTML
NE356	JE2-derived strain with a transposon in *pyrE* gene	NTML
NE1759	JE2-derived strain with a transposon in *pyrF* gene	NTML
NE1048	JE2-derived strain with a transposon in *pyrP* gene	NTML
*E. coli strains*		
DH5α	Routine laboratory strain	
BW25113	F-Δ(araD-araB)567ΔlacZ4787(::rrnB-3)rph-1Δ(rhaD-rhaB)568hsdR514	CGSC
JW0031-1	BW25113 Δ*carB*745::*kan*	(22)
*Plasmid*		
*pMK4*	*E. coli*-*S. aureus* shuttle plasmid, Cam^r^ Amp^r^	(23)

Growth dynamics was followed on 384-wells plate (Corning #3640) using a plate reader Infinite 200 Pro (Tecan) at 37°C with an orbital shaking of 5 mm. The absorbance was measured every 6 minutes at optical density 595 nm (OD_595nm_).

### *In Vitro* Exposure to H_2_O_2_

After an overnight culture, *S. aureus* was inoculated in TSB to an OD_595nm_ of 0.01 and 50 µL of the bacterial culture were dispensed on a 384-wells plate (Corning #3640) and incubated at 37°C with orbital shaking. When bacterial growth reached the early exponential phase (OD_595nm_ ~0.2-0.3), H_2_O_2_ was added at different final concentrations (0 mM, 0.00061 mM, 0.00122 mM, 0.00244 mM, 0.00488 mM, 0.00977 mM, 0.01953 mM, 0.03906 mM, 0.07813 mM, 0.156 mM, 0.313 mM, 0.625 mM, 1.25 mM, 2.5 mM, 5 mM, 10 mM, 20 mM, 40 mM, 80 mM, 160 mM and 320 mM). H_2_O_2_ 30% (Sigma-Aldrich) was freshly diluted in TSB before use. Briefly, H_2_O_2_ was diluted at 11-fold desired concentration and 5 µL was added to the 50 µL bacterial culture (total volume per wells: 55 µL). A dose-response curve based on the growth rate during exponential phase curves after H_2_O_2_ exposure was made. We used the *Doubling Time Software v1.0.10* (http://www.doubling-time.com) to estimate the growth rate (24). It allowed us to identify the highest sublethal concentration, where ~30% of bacterial growth rate of the exponential phase was affected after addition of H_2_O_2_.

### RNA Isolation, RNA-Seq and qRT-PCR

Total RNA was extracted as described previously by Fischer et al. (25). Briefly, overnight cultures were diluted to an OD_595nm_ of 0.01 and grown in fresh TSB for 3h at 37°C with constant shaking (180 rpm). Bacteria were washed and lysed with lysostaphin 25 µg/ml (Sigma) and RNA was isolated with RNeasy mini kit plus (Qiagen). gDNA was removed with DNase I digestion as previously described by Schuster et al. (26). Total RNA was quantified with Cubit or Nanodrop. RNA-Seq analysis was made as described by Mikheyeva et al. (27). Briefly, the RNA integrity was determined using Agilent 2100 BioAnalyzer (Agilent Technologies) to verify the quality of extracted RNA and one microgram of total RNA was ribo-depleted with the Ribo-Zero kit (Illumina). For the library preparation, the truseq total RNA stranded was used. Using Illumina HiSeq 4000 sequencer, oriented 50 bases single-read sequencing was performed. Finally, RNA-Seq and data analysis were carried as described in Cherkaoui et al. (28). Low quality reads and reads containing adapter and poly-N were removed and remaining reads were aligned on USA300 genome (accession number: CP000255). For qRT-PCR, total RNA was reverse transcribed using PrimerScript Reverse Transcriptase (TaKaRa) and genes were quantified using a Brilliant SYBR green master mix (Agilent). The primers used in this study are described in **Table 2**. For RNA isolated after phagocytosis, a preamplification with the TaqMan™ PreAMp Master Mix Kit (Applied Biosystems) was performed. Quantitative PCR (qPCR) reactions were performed in a Bio-Rad CFX96 and normalized using intensity levels recorded for the *hu* gene as previously described in Garzoni et al. (29).

**Table 2:** Primers.

Primers (5’ - 3’)		
Gene	Forward	Reverse
***HU***	CCTCAAAGTTACCGAAACCAA	AGCTGGTTCAGCAGTAGATGC
***carA***	TTCCAGGGATTGCAGGTGTT	AACGCCATCTGGAGCCATTG
***carB***	CAGCCACAAGGGAAAACAGC	TTCCAGGGATTGCAGGTGTT
***pyrB***	TCTGGTGAACGTCAACTACCA	TACCATCACCAGCATTCGCA
***pyrC***	CGCGCAGGCATTCATGTTAC	GTGCATGGTCTGTTGCGATAC
***pyrD***	AAACACAACGCGACAACGAG	AACGTGCGATGCCTTTGTTC
***pyrE***	CCTTTAGTTCGAGGCGCAATC	GTGACTGAAGATCCCCCTGTC
***pyrF***	GCTGCTGGTGGCGTAAAAAT	TATGCGTCGAACCAAGCTGT
***pyrG***	TTGGCGGTACAACAGGTGAT	ATGTTGCGTTGGCTTCGTTT
***pyrP***	AGCAGCGTTACTAGCTTCGG	TCCCGTGATAATTGGCGTGA
***pyrR***	TGCCGCAATACAACGTACAG	CCGTTCGACCAGTATACAGCA
**Cloning**		
	**Forward**	**Reverse**
***carA***	GGCGGGTACCAAGGAGGAACAAT CATGCAAAGCAAACGTTATCTAGTG	CGATCTGCAGTTAGGCATTGATATGACGCTCC

### Construction of Plasmid With Constitutive *carA* Expression

A polymerase chain reaction (PCR) amplification of *carA* gene was made using reverse and forward primers and JE2 chromosomal DNA as template. Forward primer contained a KpnI tail and reverse primer a Pst1 tail. A *Pfu* DNA polymerase (Promega) was used for PCR products amplification. Fragments were ligated using KpnI and Pst1 enzymes in a custom designed pmk4-based vector containing a P*glyS* promoter. The resulting plasmid was electroporated into the non-restrictive *S. aureus* strains RN4220 prior to transfer to *carA* mutant strains. Complemented strains were selected with chloramphenicol 10 µg/ml. The construction was sequenced and verified. Restoration of the *carA* function was confirmed by growth kinetic and by PCR of *carA* expression.

### EdU Click Labeling of Newly Replicated DNA in *S. aureus*

We monitored DNA synthesis as described in Martel et al. (30). Briefly, overnight cultures of *S.aureus* were diluted to OD_595nm_ of 0.01 in 30 ml TSB and grown at 37°C with agitation until OD_595nm_ of 0.2 was reached. The culture was separated in 12 samples of 2ml. EdU from Click-iT EdU^®^ assay (Invitrogen) was added (final concentration 0.12 mM) and incubated for 15 minutes at 37°C. A control group was left untreated to assess the unspecific coloration of the Alexa Fluor^®^-azide. H_2_O_2_ was added at a final concentration of 20 mM to the H_2_O_2_ treated group. Bacteria were grown for an additional 30 minutes at 37°C with shaking and growth was stopped by fixation with 90% methanol. Fixed bacteria were washed with 1.5 ml PBS and permeabilized with 200 μL lysostaphin (80 μg/mL in TE buffer) and incubated 15 minutes at 37°C as described in Rodriguez and Kuehn (31). EdU incorporation was revealed by the adjunction of 200 µl of Click-iT^®^ reaction cocktail with Alexa Fluor488^®^-azide prepared according to the manufacturer’s instructions. After 30 minutes of incubation at room temperature, bacteria were washed with PBS and resuspended in 1.5ml of filtered PBS with 1% human serum albumin. Five µl of 200µl/ml DAPI (Applichem) was added to the samples and incubated at room temperature for 10 minutes. Samples were visualized by a Gallios Flow Cytometer (Beckman Coulter) set for 488 nm (laser) with a 525/40 filter with 30’000 events per samples. Data were analyzed with Kaluza Analysis Software (Beckman Coulter). DAPI-negative events were not considered as bacterial cells and excluded. The percentage of cells with incorporated EdU and the arithmetic mean of fluorescence of EdU-positive cells were reported to the percentage of corresponding events in the untreated condition to compare the different experiments.

### Isolation of Human Neutrophils

Human neutrophils were purified from 10 ml citrated blood samples of healthy donors after obtaining their informed consent. Total blood cells were separated by sequential Ficoll-Hypaque differential density centrifugation as described in Genestet et al. (32). Neutrophils in the pellet were resuspended in phosphate-buffered saline (PBS) solution. Erythrocytes were lysed twice with a frozen hypotonic lysis buffer as described by Dri et al. (33). Briefly, the lysis buffer contained 155 mM NH_4_Cl, 10 mM KHCO_3_, 0.1 mM EDTA for a pH of 7.4. After erythrocytic lysis, cells were centrifugated for 10 minutes at 460 g at 4°C and neutrophils were resuspended in Ca^+2^ and Mg^+2^ free HEPES-buffered saline (HBS) solution containing 140 mM NaCl, 5 mM glucose, 5 mM KCl, 5 mM HEPES and 0.2% of bovine serum albumin (BSA). Cells were kept on ice until use. Directly before use, cell suspensions were supplemented with 1 mM CaCl_2_ and 1 mM of MgCl_2_.

### Microbicidal Activity Assay With Human Neutrophils

The microbicidal activity of neutrophils was assessed according to the method described in Decleva et al. (34). In brief, 4x10^6^ neutrophils/ml were incubated at 37°C with shaking 160 rpm with serum-opsonized *S. aureus* at a bacteria/neutrophil ratio of 3:1. After one hour of incubation, aliquots were diluted 50 times in water with NaOH (pH 11) for 5 minutes in order to lyse neutrophils. Then, tubes were vortex and diluted into 0.9% NaCl solution and plated on Petri dishes. The next day, CFU were counted and the percentage of killing was calculated according to the number of CFU at T0.

### Statistics

GraphPad Prism 8 for Windows (GraphPad Software, San Diego, USA) and Rstudio 1.2.5001 (Rstudio, Boston, USA) were used for data processing, graph plotting and statistical analysis. Pairwise comparisons were performed to detect differentially expressed transcripts between H_2_O_2_ treated and control condition and statistical significance (False Discovery rate < 0.05) was determined using DeSeq2 3.10 (available at http://www.bioconductor.org/packages/release/bioc/html/DESeq2.html). For the IC_50_ analysis and curve fit, a normalized log(response) inhibition model was used. Where Y = growth rate (percentage), X = H_2_O_2_ concentration, IC_50_ = H_2_O_2_ concentration that decrease growth rate from 50%. Kruskal-Wallis test with Dunn’s multiple comparison tests were applied to compare the EdU labeling incorporation in newly replicated DNA.

## Results

### Hydrogen Peroxide Significantly Affects the Expression of Genes Involved in Pyrimidine Metabolism

**Figure 1:**
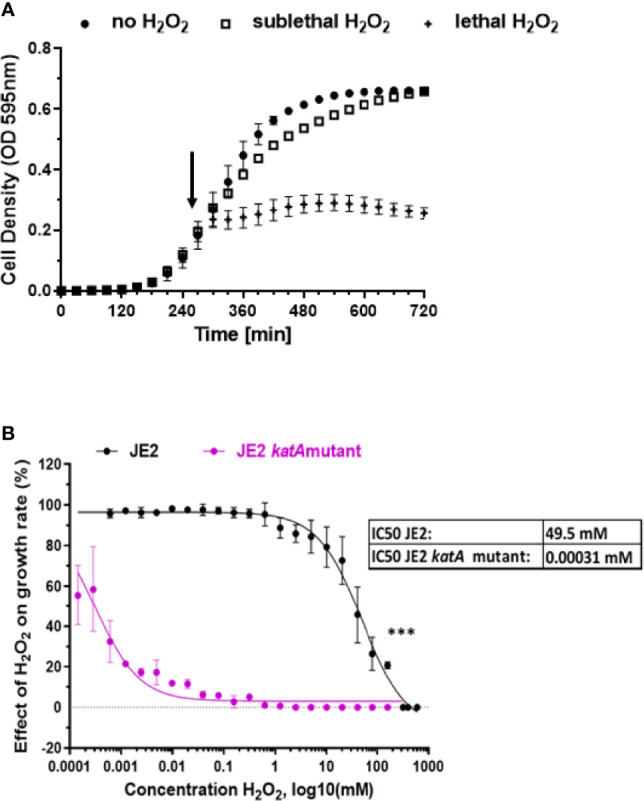
Effect of H_2_O_2_ on *S. aureus* growth. **(A)** *S. aureus* wild-type JE2 strain growth in absence of H_2_O_2_ (●) and in the presence of two H_2_O_2_ selected concentrations: sublethal (20mM) (□) or lethal (160 mM) (+). H_2_O_2_ was added in the early exponential phase, as indicated by an arrow. **(B)** Effect of 22 different concentrations of H_2_O_2_ on wild-type *S. aureus* JE2 strain and its corresponding *katA* mutant and compared to growth in the absence of H_2_O_2_. Normalized values were expressed as percent of growth in the absence of H_2_O_2_ on the same experiment. Results represent the mean ± SD of three independent experiments (four replicates for each H_2_O_2_ concentration). A nonlinear regression model was applied to estimate the IC_50_. ***p < 0.001.

We first investigated growth of *S. aureus* JE2 strain at different H_2_O_2_ concentrations (**Figure 1**). H_2_O_2_ was added at the early exponential phase (OD_595nm_ 0.25) and the effect on growth dynamic was observed during the entire exponential phase (**Figure 1A**). We tested 22 different concentrations of H_2_O_2_, ranging from 0.61 µM to 320 mM and we calculated the growth rate during the exponential phase after addition of the respective amounts of H_2_O_2_. Based on these results, the apparent IC_50_ of H_2_O_2_ for JE2 growth was 49.5 mM (**Figure 1B**). 20 mM H_2_O_2_ is the highest concentration which did not affect growth in a significant statistical manner (p=0.053). Thus, we defined 20 mM H_2_O_2_ as the highest sublethal concentration affecting moderately bacterial growth. This concentration was used for most experiments shown below. In order to verify that the growth of *S. aureus* is indeed affected by H_2_O_2_, we tested similar H_2_O_2_ concentrations on a catalase-deficient strain (JE2 *katA* mutant). Catalase is an enzyme that metabolizes H_2_O_2_ into O_2_ and H_2_O. In the absence of catalase expression, *S. aureus* is approximatively 10^7^ times more susceptible to H_2_O_2_ killing with an estimated IC_50_ of 0.31 µM (**Figure 1B**).

**Figure 2:**
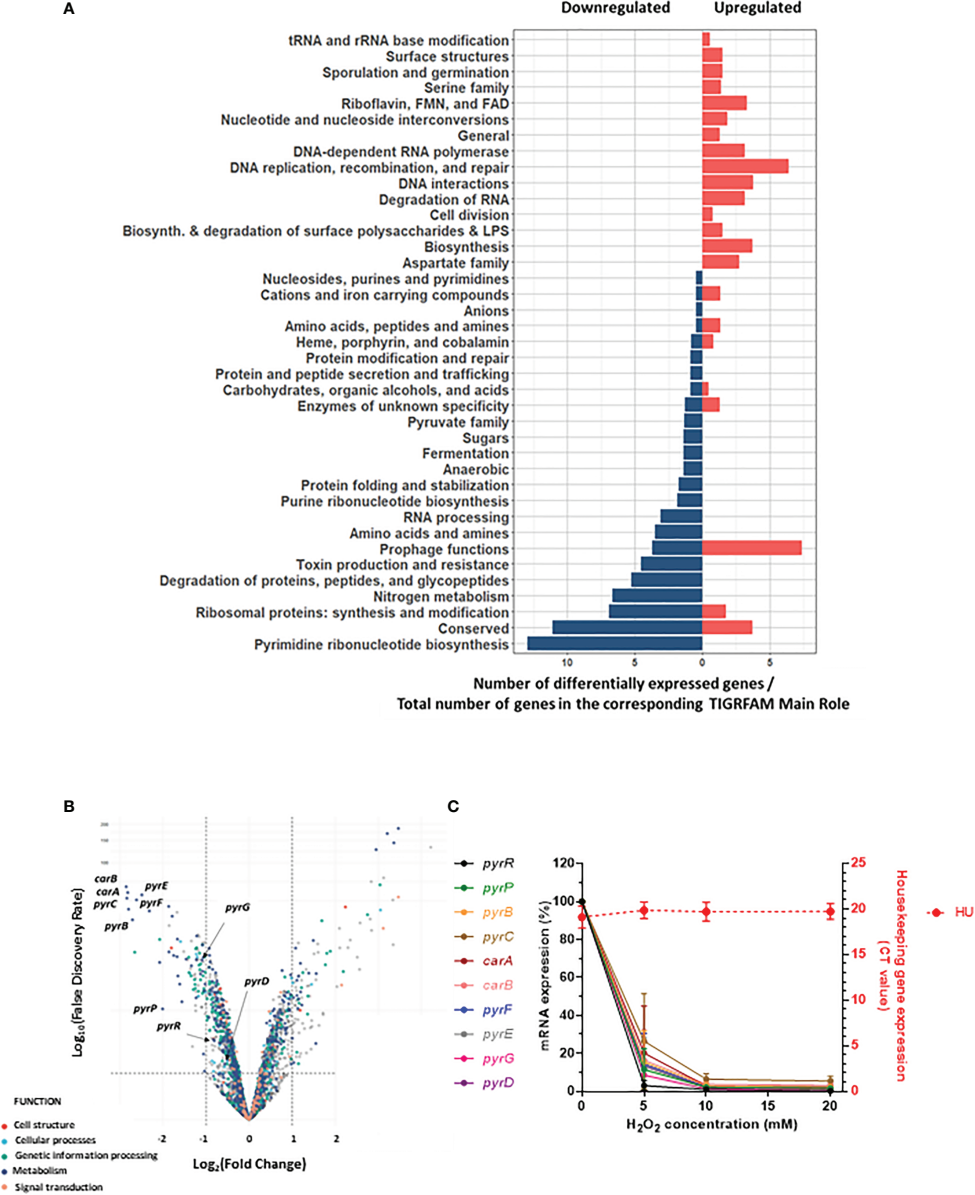
Downregulation of genes involved in pyrimidine metabolism after H_2_O_2_ exposure. **(A)** Pyramid plot displaying significant affected *S. aureus* genes in the presence of H_2_O_2_. From the total number of genes present in the TIGRAM main role category, the significant affected gene categories were selected, based on TIGRFAM category “Sub-Role” (FDR <0.05 and fold change ≤-2 or ≥2). Gene categories upregulated or downregulated upon H_2_O_2_ exposure are indicated in red or blue, respectively. **(B)** Volcano plot of RNA-Seq data representing the differential gene expression analysis of *S. aureus* JE2 exposed or not to one hour of sublethal H_2_O_2_. The upper left panel represents genes significantly (FDR <0.05 and Fold change ≤-2) downregulated after H_2_O_2_ exposure. The upper right panel represents genes significantly (FDR <0.05 and Fold change ≥2) upregulated after H_2_O_2_ exposure. RNA-Seq results were normalized using DESeq2 software. **(C)** Effect of different concentrations of H_2_O_2_ on *pyr* genes expression. Bacteria were exposed for one hour to different concentrations of H_2_O_2_. *pyr* gene expression was assessed by qRT-PCR. Relative expression levels were determined by comparing cycle threshold (CT) of each gene to the CT value of the *hu* gene for the same cDNA preparation. Left y axe represents mRNA expression of *pyr* genes and right y axe shows the minimal variation of *hu* CT values with different H_2_O_2_ concentrations (CT 20 +/- 0.3). These results represent the mean ± SD of three independent experiments (three replicates for each H_2_O_2_ concentration and three replicates for each gene).

The impact of sublethal H_2_O_2_ concentration on gene expression in *S. aureus* was addressed by RNA-Seq. H_2_O_2_ was added at the early exponential phase and after one hour of exposure to 20 mM (or 0 mM for control conditions), total bacterial RNA was extracted and their respective transcriptome was determined. This time point was chosen in order to study bacterial response after the immediate stress response such as catalase expression. **Figure 2** illustrates the differential expression analysis for *S. aureus* treated with H_2_O_2_ *versus* untreated. We observed 190 differentially expressed genes with a fold change ≤ - 2 and ≥ 2 with statistical significance (False Discovery Rate (FDR)). Among these genes, 98 were upregulated and 92 downregulated (**Table 1** and **Supplementary Data**). Genes with unknown function (n=85, 44.7%) were removed from **Table 1**. In order to identify gene categories affected by the presence of H_2_O_2_, we analyzed RNA-seq data based on TIGRFAM gene categories. **Figure 2A** illustrates the number of differentially expressed genes on the total number of genes in the corresponding TIGRFAM main role. TIGRFAM is a protein families database designed to support genome annotation (35, 36). Only genes with expression that were statistically (FDR<0.05) and significantly changed (fold change ≤-2 or ≥2) were analyzed. Pathways including DNA replication, recombination and repair and DNA interactions had the highest number of genes upregulated, followed by riboflavin, FMN and FAD pathways. The upregulated genes belonged to various families, including genes involved in riboflavin metabolism [typically involved in redox response (37)], phage genes, genes involved in DNA repair, as well as several gene encoding non-identified hypothetical proteins. The downregulated genes belonged to different gene families, including degradation of proteins, peptides, and glycopeptides, ribosomal proteins or toxin production and resistance. Interestingly, the category of genes involved in pyrimidine ribonucleotide biosynthesis was the pathway with the most important downregulation. There was an almost complete downregulation of the different genes involved in the pyrimidine ribonucleotide biosynthesis (fold changes ranging from -7 to -2) (**Figure 2B**). We confirmed a dose-dependent downregulation observed in the RNA-Seq by qRT-PCR (**Figure 2C**). We decided to focus our research on this gene family.

### Inactivation of *carA* and *pyrP* Genes Affects *S. aureus* Growth

**Figure 3:**
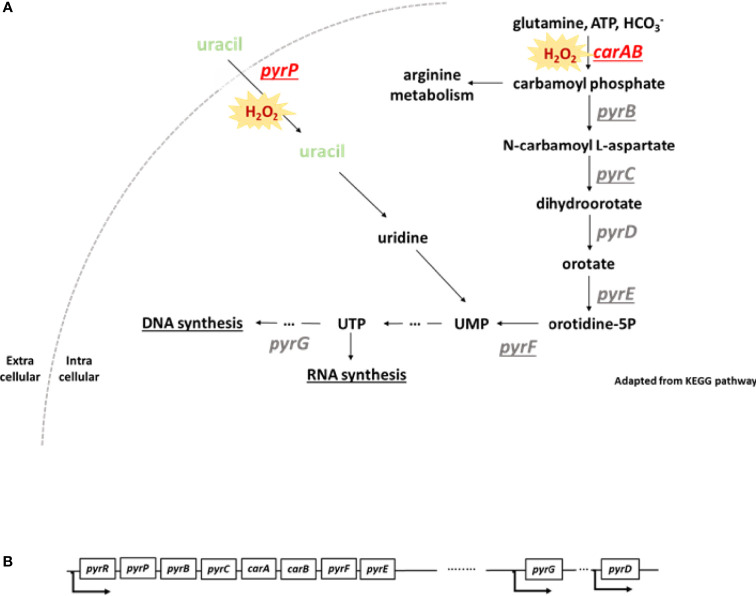
*pyr* operon and scheme of pyrimidine biosynthesis pathway. **(A)** Under oxidative stress, genes involved in pyrimidine biosynthesis are downregulated. Loss of function of *carA* and *pyrP* leads to an alteration of bacterial fitness. Pyrimidine metabolism is divided in two different pathways: the *de novo* and the salvage pathways. The *de novo* pathway uses glutamine, ATP and bicarbonate $\left(\text{HC}{\text{O}}_{3}^{-}\right)$ in the cell to produce uridine monophosphate (UMP). The salvage pathway uses extracellular uracil to generate UMP. Loss of function of *carA* and *pyrP* genes leads to an alteration of bacterial fitness and a higher sensitivity to H_2_O_2_. UMP, uridine monophosphate, UTP, uridine triphosphate. The underlined genes are located on the same operon. This scheme was adapted from KEGG pathway. **(B)** Some of the *pyr* genes are located on an operon and transcribed from a single promoter. *pyrG* and *pyrD* are located further in the genome and transcribed with their own promoter.

Next, we addressed the impact of inactivation of *pyr* genes on the fitness of *S. aureus*. To study this question, we used a library of mutant strains, the Nebraska Transposon Mutant Library (NTML) (38, 39), to address the question of inhibition of the pyrimidine pathway. We used six mutants of the pyrimidine biosynthesis pathway: *carA*, *pyrB*, *pyrD*, *pyrE*, *pyrF*, *pyrP*. The biosynthesis of pyrimidine is divided into two different pathways: i) the *de novo* pathway, which uses glutamine, ATP and bicarbonate for uridine monophosphate (UMP) synthesis and, ii) the salvage pathway, which uses extracellular uracil to synthetize UMP (**Figure 3A**). Several *pyr* genes such as pyrP, *pyrB*, *pyrC*, *carA*, *pyrF* and *pyrE* are located on the same operon and transcribed from a single promoter (**Figure 3B**).

**Figure 4:**
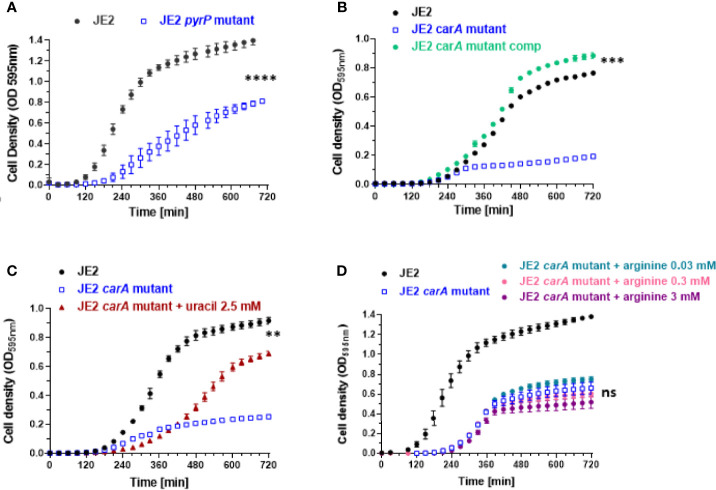
Effect of *carA* and *pyrP* mutations on bacterial growth and partial rescue of *carA* mutant with uracil and *carA* complementation. **(A, B)** Bacterial growth analysis of wild-type *S. aureus* JE2 strain, *pyrP* and *carA* mutants (from the Nebraska transposon mutant library) and *carA* mutant carrying a multicopy plasmid expressing *carA* (JE2 *carA* mutant comp). **(C)** Partial growth restoration of *carA* mutants after supplementation of 2.5 mM of uracil in the medium. **(D)** Absence of growth restoration with different arginine concentrations. Results represent the mean ± SD of three independent experiments (four replicates for each strain). **p < 0.005, ***p < 0.001, ****p < 0.0001, ns p > 0.005 by Mann-Whitney test.

We first investigated the growth of *pyr* mutants and we observed two mutants displaying a growth defect. Growth was strongly affected for *carA* and *pyrP* mutants (**Figures 4A, B**). The growth of *pyrB*, *pyrD*, *pyrE*, *pyrF* mutants was not affected (**Figures 1A–D** and **Supplementary Data**).

**Figure 5:**
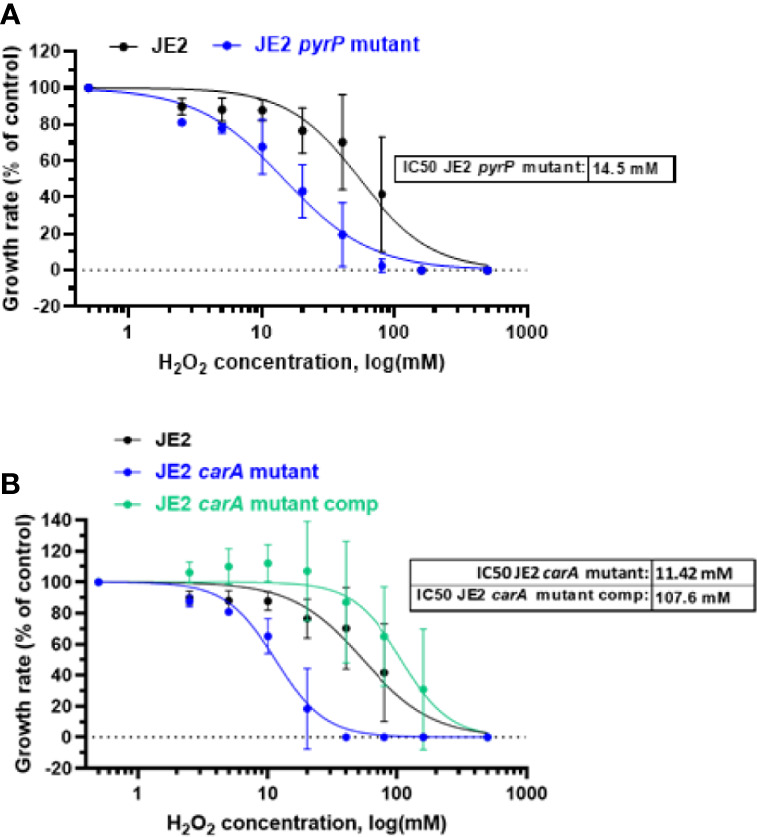
H_2_O_2_ effect on bacterial growth rate. Growth of wild-type *S. aureus* JE2 strains and *pyrP* **(A)** and *carA* mutants and *carA* mutant carrying a multicopy plasmid expressing *carA* (JE2 *carA* mutant comp) **(B)** was analysed in the presence of 8 different concentrations of H_2_O_2_ and compared to growth in the absence of H_2_O_2_. Normalized values were expressed as percent of growth in the absence of H_2_O_2_ on the same experiment. Results represent the mean ± SD of two independent experiments (four replicates for each H_2_O_2_ concentration). A nonlinear regression model was made to estimate the IC_50_. p > 0.005.

To analyze the effect of H_2_O_2_ on each *pyr* mutant, we exposed each mutant to eight different H_2_O_2_ concentrations, ranging from 2.5 mM to 160 mM of H_2_O_2_ for one hour and observed the effect on their growth rate. The growth rate was normalized according to the growth rate in absence of H_2_O_2_ and compared with the parental strain. The estimated IC_50_ for each mutant is represented on each graph. *pyrB*, *pyrD*, p*yrE* and *pyrF* mutants have a H_2_O_2_ sensitivity close to the parental strain with an IC_50_ respectively at 80.5 mM, 73.5 mM, 72.7 mM and 46.7 mM respectively (**Figure 2** and **Supplementary Data**). In contrast, *pyrP* and *carA* mutants were more sensitive to H_2_O_2_ than the parental strain with IC_50_ estimated to 14.5 mM and 11.4 mM (**Figures 5A, B**).

Among all *pyr* mutants found in the NTML, we observed that loss of function of two *pyr* genes, *carA* and *pyrP*, induced a drastic alteration of bacterial fitness. The *carA* gene encodes for the small subunit of the carbamoyl phosphate synthase, a subunit of a large enzymatic complex associated with CarB, initiating the first reaction in pyrimidine and arginine metabolism (40) (**Figure 3A**). The *pyrP* gene encodes for an uracil permease located in the bacterial membrane, which transports uracil into the cell (41) (**Figure 3A**). Given the strong phenotype of the *carA* mutant, showing important growth defect and an increased sensitivity to H_2_O_2_ in *S. aureus*, we focused our investigations on this strain.

### *carA* Deletion Affects Growth and Sensitivity to H_2_O_2_

To demonstrate that *carA* gene mutation is involved in growth and sensitivity to H_2_O_2_, we first complemented the *carA* mutant with a pMK4 multicopy plasmid carrying *carA* gene under the control of the PglyS constitutive promoter (42, 43) (see materials and methods). Bacterial growth and sensitivity to H_2_O_2_ was further analyzed. CarA complementation restores *carA* mutant growth, similar to wild-type JE2 strain (**Figure 4B**). Moreover, the complemented strain showed a strong decreased sensitivity to H_2_O_2_ with an IC_50_ of 107.6 mM, compared to the IC_50_ of the *carA* mutant (IC_50_ = 11.42 mM) (**Figure 5B**). These results show that loss of function of *carA* gene not only affects bacterial growth but also plays a role in the sensitivity to H_2_O_2_.

### Uracil Restores Growth Defect in *carA* Mutant

As uracil is a common and natural pyrimidine derivative, we supplemented the culture medium with uracil. As depicted in **Figure 4C**, 2.5 mM uracil led to a substantial recovery of growth of the *carA* mutant. We tested higher concentrations of uracil, but they did not further enhance bacterial growth and even had a deleterious effect on growth above 5 mM (data not shown). Note that the recovery was delayed and incomplete suggesting that this compensatory mechanism might be saturated and does not fully compensate for the loss of the *carA* gene. *carA* is also involved in arginine metabolism. We therefore tested different concentrations of arginine and did not observe any growth restoration (**Figure 4D**).

### H_2_O_2_ Induces a Decreased DNA Replication in *S. aureus*

**Figure 6:**
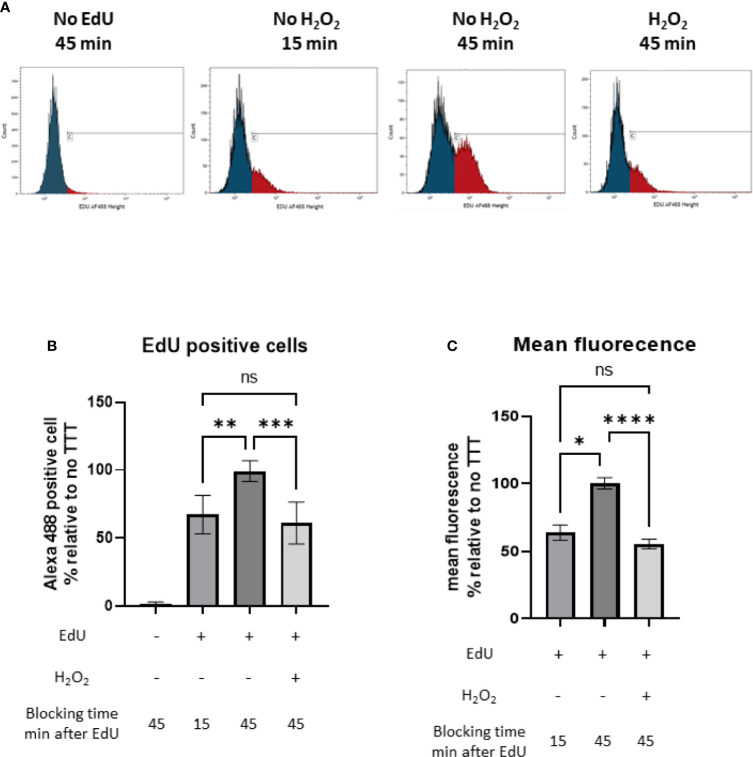
Monitoring of the DNA synthesis by following the incorporation of the nucleotide analog EdU (5-ethynyl-2′-deoxyuridine), Click-iT^®^ assay (Invitrogen) with and without H_2_O_2_ treatment. **(A)** Number of events under fluoresence intensity. Blue and red peaks represent respectively unspecific binding of Alexa Fluor488^®^-azide (defined by the control without EdU) and bacteria with active DNA replication. **(B)** Percentage of EdU positive *S. aureus* normalized to the untreated 45 min condition. **(C)** Arithmetic mean fluorescence of EdU positive *S. aureus* normalized to the untreated 45 min condition. These results represent mean ± SD of three different experiments (three replicates for each condition). ns, not significant, *p < 0.05, ** p < 0.005, ***p < 0.001, ****p < 0.0001 by Kruskal-Wallis test with Dunn’s multiple comparison tests.

We then wanted to study if the downregulation in pyrimidine metabolism observed after addition of sublethal concentration of H_2_O_2_ was due to a decreasedbacterial DNA replication. We performed an EdU-Click labeling assay and compared the incorporation of the nucleotide analog 5-ethynyl-2’-deoxyuridine (EdU) in *S. aureus* JE2 with and without 20 mM of H_2_O_2_ by flow-cytometry (**Figure 6**). H_2_O_2_ was added after EdU to avoid oxidation. EdU untreated *S. aureus* showed some unspecific binding of the Alexa Fluor488^®^-azide. In the absence of H_2_O_2_, EdU was actively incorporated in DNA over time while in the H_2_O_2_ treated group, we did not observe EdU incorporation after H_2_O_2_ treatment (**Figure 6A**). The percentage and the arithmetic mean fluorescence of EdU positive *S. aureus* were normalized to the EdU H_2_O_2_ untreated condition. We observed that, in presence of 20 mM of H_2_O_2_, DNA replication was limited while incorporation of EdU was increased by approximately 40% in the absence of H_2_O_2_ (**Figures 6B, C**). These results suggest that the effect H_2_O_2_ on genes involved in pyrimidine metabolism leads to a decreased DNA replication.

### *carA* Gene Is Not Involved in Survival After Neutrophil Phagocytosis

**Figure 7:**
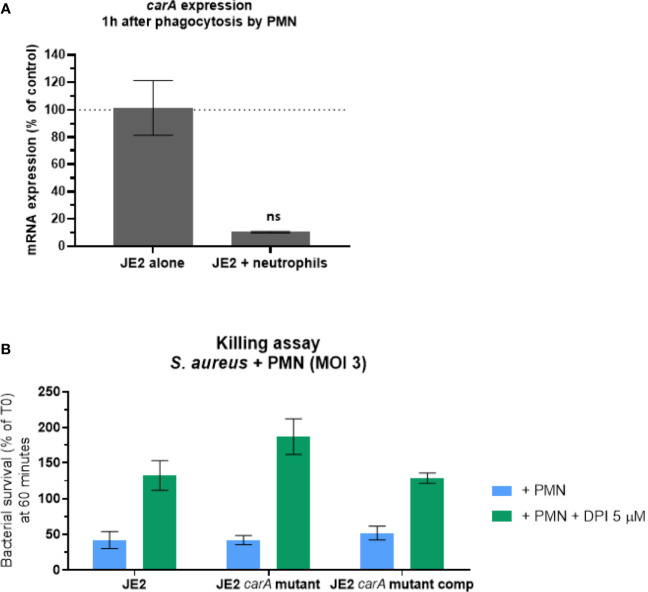
*carA* expression and *S. aureus* survival 60 minutes after phagocytosis by human neutrophils. **(A)** Expression analysis of *carA* with qPCR one hour after phagocytosis by human neutrophils. Gene expression was normalized with the gene expression in non-phagocytosed bacteria. These results represent the mean ± SD of two independent experiments. **(B)** Bacterial survival was estimated after 60 minutes of co-culture with human neutrophils with a multiplicity of infection (MOI) of 3. ROS production inside the phagosome was either conserved (blue) or inhibited (green) by 5 µM of diphenyleneiodonium (DPI). CFU counts following the experiment were normalized according CFU count from the initial inoculum. These results represent the mean ± SD of three independent experiments. ns, not significant, p>0.05 by Kruskal-Wallis test.

Due to the increased sensitivity of *carA* mutant to H_2_O_2_, we investigated the impact of the lack of *carA* expression in a more complex model than *in vitro* H_2_O_2_ exposure. We first analyzed the expression of *carA* gene after one hour of phagocytosis (**Figure 7A**) and observed downregulation similarly to what we observed *in vitro*. Then, we assessed the survival of JE2, *carA* mutant and *carA* complemented strains after phagocytosis by human neutrophils. Opsonized *S. aureus* strains were incubated with freshly isolated human neutrophils for 1 hour and their survival was measured by counting the colonies on agar plate after 24 hours, in presence or absence of an irreversible NOX2-inhibitor (DPI). The number of CFUs was normalized according the CFUs number at T0. We observed that in presence of neutrophils, the survival rate dropped around 40% for all the three strains (**Figure 7B**). The role of the ROS generating NOX2 was confirmed by the fact that the killing activity of neutrophils was inhibited by 5 µM of DPI. Therefore, the absence of *carA* expression did not affect the bacterial survival after phagocytosis in presence and in absence of ROS.

## Discussion

The aim of this study was to understand why ROS are crucial in the host defense against *S. aureus*. We observed important changes in gene expression at sublethal H_2_O_2_ concentrations, in particular a concerted downregulation of enzymes of pyrimidine metabolism. This downregulation was caused by a decreased in DNA replication and was strongly associated with decreased extracellular, but not intracellular survival of the bacteria. We hypothesize that this is a long-term survival mechanism of *S. aureus*: avoiding growth of extracellular *S. aureus* which can cause severe infection and lead to death of its host, while assuring its intracellular survival.

After one hour of exposure to a sublethal concentration of H_2_O_2_, the number of upregulated and downregulated genes was approximately equal, suggesting that under our experimental conditions, H_2_O_2_ does not lead to unspecific toxicity, but rather induces a cellular response. Interestingly among upregulated genes, the expression of genes involved in antioxidant response was unchanged, most likely because they are expressed within minutes after the onset of the oxidative stress and already decreased to normal levels one hour after addition of H_2_O_2_. Our hypothesis was that H_2_O_2_ affects *S. aureus* general fitness and we decided to focus on downregulated genes. Among downregulated genes, we observed that most of the genes involved in pyrimidine biosynthesis were strongly affected in *S. aureus* following one hour of exposure to a sublethal concentration of H_2_O_2_. We identified two genes in this family, *carA* and *pyrP*, that were important for bacterial growth in rich medium and H_2_O_2_ sensitivity. We observed impaired DNA replication after H_2_O_2_ exposure. This decrease in DNA replication and transcription was dependent on the downregulation of pyrimidine biosynthesis. This concerted response represents a compensation mechanism due to the presence of H_2_O_2_ ensuring bacterial survival. Indeed, similar amount of H_2_O_2_ resulted in bacterial death of a *carA* mutant, a non-compensable mutation. We then confirmed that the expression of *carA* was also decreased after one hour of phagocytosis by human neutrophils. Furthermore, the fitness of the *carA* mutant was particularly affected and could be reversed by genetic rescue. Yet, survival of *carA* mutant after phagocytosis by human neutrophils was not different from the parental strain.

Pyrimidine metabolism leads to the formation of pyrimidine nucleotides (namely uracil, cytosine and thymine), which are used for nucleic acid synthesis (44), energy production (UTP or CTP) and other key cellular functions (45). Both *carA* and *pyrP* are involved in the first step of two pathways, *de novo* and salvage pathways, and, unlike the other genes in the pathway, they are crucial, and no alternative pathway can substitute their function. We think that in nutrient rich media, the salvage pathway is working, as bacteria are able to grow, however it is less efficient than the *de novo* synthesis pathway, explaining why the growth of the *carA* mutant is delayed (but not inhibited as can be seen in minimal media).

It has been observed that *carA* plays an important role in virulence in other bacterial species, such as *Pseudomonas syringae* (46), *Escherichia coli* (47), *Xanthomonas citri* (48) and *Francisella tularensis* (49). Similar to the genomic organization characterized in *B. subtilis*, several *pyr* genes (*pyR*, *pyrP*, *pyrB*, *pyrC*, *carA*, *carB*, *pyrF* and *pyrE*) of *S. aureus* are located on an operon and transcribed from a single promoter (**Figure 3B**). The transcription of the operon is negatively regulated by the binding of PyrR to specific anti-termination sites [described in detail in Turnbough and Switzer (50)]. PyrR peptide sequence does not contain cysteines redox-sensitive residues (44). However, two known redox-sensitive transcription factors, MgrA and SarZ, are known to negatively regulate expression of pyrimidine genes under oxidative stress (9, 51). Both regulators have a direct effect on *pyrR* but MgrA also controls the expression of genes involved in biosynthesis of another *pyr* gene that are not located on *pyr* operon (51), such a *pyrG*, which was also downregulated in our transcriptomic analysis. This finding supports a potential role of MgrA through an additional transcription factor. The specific redox regulation of MgrA and SarZ effect on the downregulation of genes involved in pyrimidine biosynthesis pathway could be specifically addressed using redox biochemical approach, such as the biotin switch assay (52).

Nucleotide clumping of the bacterial genome has been described as another type of bacterial response to oxidative stress (53). This response is regulated by MgrA (53) and participates to bacterial stress tolerance by protecting the genome against ROS. Interestingly, the effect of H_2_O_2_ on genes involved in pyrimidine biosynthesis pathway is not specific to *S. aureus* and a study in *E. coli* demonstrated that H_2_O_2_ also induces a downregulation in genes involved in nucleotides and ribonucleotides production process (54).

Intriguingly, we observed a downregulation in *carA* expression following intraphagosomal oxidative stress, but we did not observe any difference in the survival of *carA* mutant and the parental strain, in the presence or absence of intraphagosomal ROS. Two aspects of this difference are relevant for this discussion: i) the underlying biochemical mechanism, and ii) the impact on the *S. aureus* reservoir for long-term survival as observed in numerous chronic infections.

A possible biochemical explanation for the decreased expression of *carA* in intracellular survival would be a high level of pyrimidine or pyrimidine precursors, in particular uracil, within the phagosome. To the best of our knowledge, phagosomal concentrations of the respective metabolites have not been measured. Another explanation would be changes in metabolism of intracellular bacteria leading to a decreased catabolism of pyrimidine or pyrimidine precursors. A third aspect that might contribute to the decreased importance of *carA* for phagosomal survival might be the decreased growth rate of intracellular *S. aureus* leading a decreased requirement of nucleotide precursors. This type of behavior has been observed during prolonged residency of *S. aureus* in non-phagocytic cells (55).

In summary, we demonstrated a novel and selective mechanism allowing *S. aureus* to survive in the presence of ROS, namely a coordinated downregulation of the pyrimidine biosynthesis pathway and a decreased in DNA replication. This downregulation has the interesting feature to selectively interfere with the growth of extracellular, but not intracellular *S. aureus*. Given this dichotomy, our results suggest that *S. aureus* is not a naive victim of host defenses in this situation but has developed an evolutionary survival strategy by modulating growth rate. Future studies should investigate the mechanisms described here in clinical strains. In our study where RNA-Seq data needs to be interpreted in the context of the entire genome, the choice of the JE2 strain was pertinent and allowed to uncover this novel *S. aureus* survival strategy. The importance of this “go unnoticed” strategy may contribute to the high mortality of CGD patients following *S. aureus* infection [at least if untreated (7)] and contribute to their long-term epidemiological success in the numerous people carrying *S. aureus*.

## Data Availability Statement

The datasets presented in this study can be found in online repositories. The names of the repository/repositories and accession number(s) can be found below: https://www.ebi.ac.uk/ena, PRJEB43496.

## Ethics Statement

Ethical review and approval was not required for the study on human participants in accordance with the local legislation and institutional requirements. The patients/participants provided their written informed consent to participate in this study.

## Author Contributions

HB, AL, VJ, PF, and KK designed experiments. HB, MR, AR, EB, FL, MM, and MS performed experiments. HB, VJ, FL, PF, and KK interpreted the results. NG performed bioinformatics analysis. HB and VJ wrote the manuscript. VJ, MS, JS, PF, and KK revised the manuscript. All authors contributed to the article and approved the submitted version.

## Funding

This research was supported by the Swiss National Science Foundation program.

## Conflict of Interest

The authors declare that the research was conducted in the absence of any commercial or financial relationships that could be construed as a potential conflict of interest.

## Publisher’s Note

All claims expressed in this article are solely those of the authors and do not necessarily represent those of their affiliated organizations, or those of the publisher, the editors and the reviewers. Any product that may be evaluated in this article, or claim that may be made by its manufacturer, is not guaranteed or endorsed by the publisher.
