# Supplementary figures and images for: Hydrogen Peroxide Affects Growth of S. aureus Through Downregulation of Genes Involved in Pyrimidine Biosynthesis
Source: Front Immunol. 2021 Sep 7;12:673985. doi: 10.3389/fimmu.2021.673985 (PMC8454235; doi:10.3389/fimmu.2021.673985)

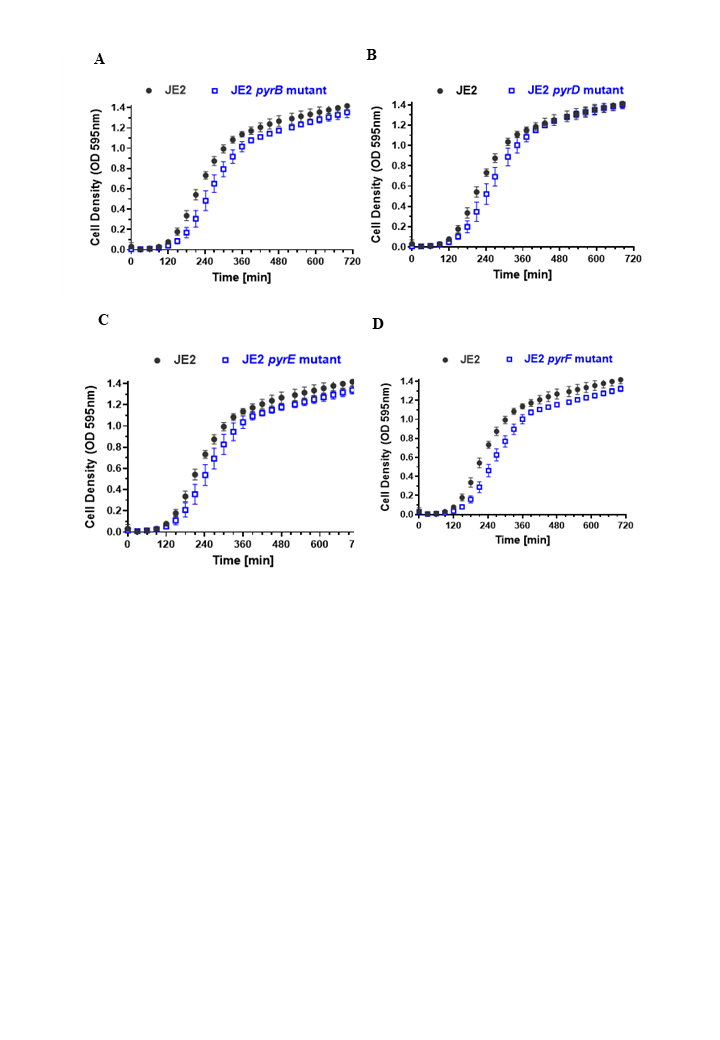

Supplement: Supplementary file 1 [file Image_1.tif]

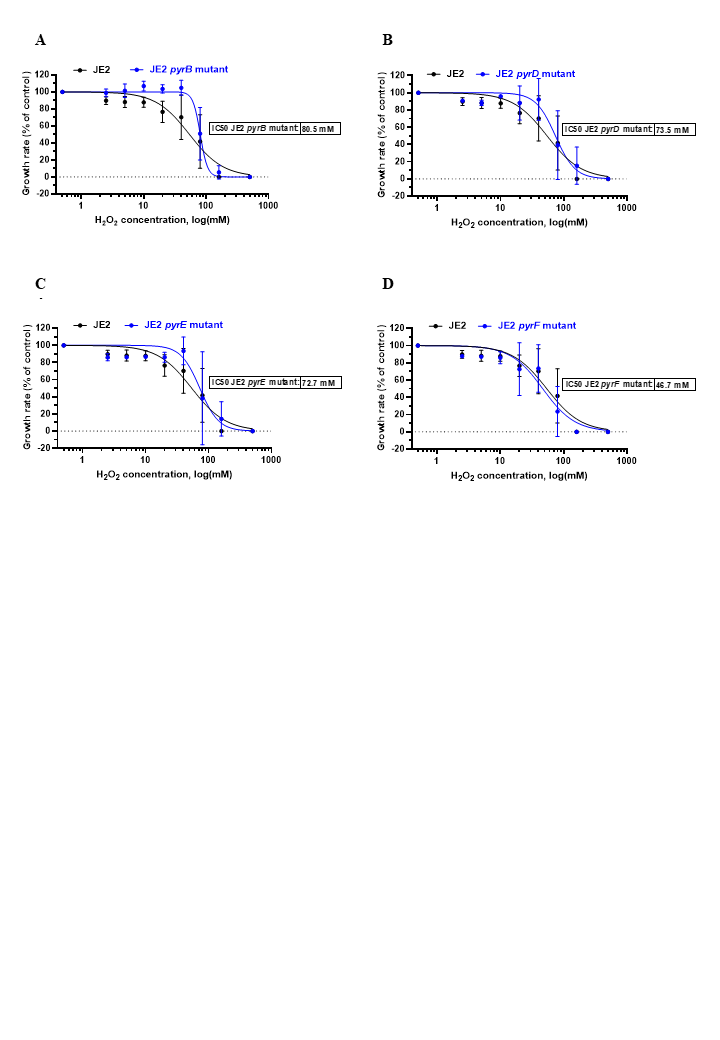

Supplement: Supplementary file 2 [file Image_2.tif]
