# Supplementary material for: Hydrogen Peroxide Affects Growth of S. aureus Through Downregulation of Genes Involved in Pyrimidine Biosynthesis
Source: Front Immunol. 2021 Sep 7;12:673985. doi: 10.3389/fimmu.2021.673985 (PMC8454235; doi:10.3389/fimmu.2021.673985)
Supplement: Supplementary file 3 [file Table_1.docx]

Supplementary Material

# Supplementary Figures and Tables

**
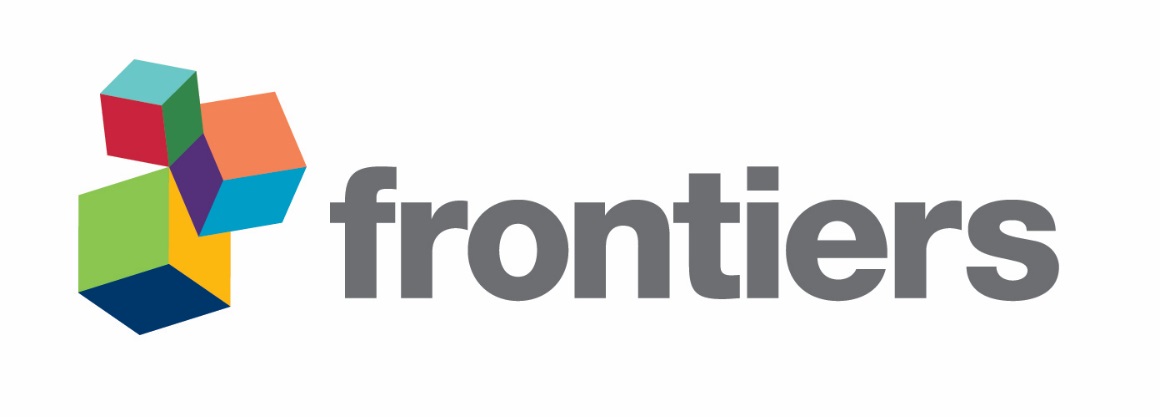
**

**Supplementary Table 1.** Up and downregulated genes after H_2_O_2_ exposure.

|  | **Upregulated genes** | | | |
| --- | --- | --- | --- | --- |
| **Locus tag** | ***Gene*** | **log2FoldChange** | **FDR** | **TIGRFAM Sub Role** |
| SAUSA300_1642 | *-* | 1.03 | 1.16E-08 | Amino acids, peptides and amines |
| SAUSA300_2538 | *-* | 1.11 | 6.15E-12 | Amino acids, peptides and amines |
| SAUSA300_0784 | *-* | 1.39 | 1.33E-13 | Amino acids, peptides and amines |
| SAUSA300_2414 | *-* | 1.13 | 4.38E-07 | Aspartate family |
| SAUSA300_1286 | *-* | 1.15 | 1.87E-08 | Aspartate family |
| SAUSA300_1249 | *-* | 1.19 | 2.77E-16 | Fatty acid and phospholipid metabolism |
| SAUSA300_1004 | *-* | 1.18 | 7.41E-07 | Biosynthesis and degradation of surface polysaccharides and lipopolysaccharides |
| SAUSA300_1221 | *-* | 1.55 | 1.19E-03 | Carbohydrates, organic alcohols, and acids |
| SAUSA300_2139 | *-* | 1.01 | 4.95E-04 | Cations and iron carrying compounds |
| SAUSA300_0068 | *-* | 1.04 | 1.66E-02 | Cations and iron carrying compounds |
| SAUSA300_1005 | *-* | 1.08 | 1.70E-21 | Cations and iron carrying compounds |
| SAUSA300_1961 | *-* | 3.05 | 7.30E-43 | Cell division; Chromosome-associated proteins |
| SAUSA300_0811 | *-* | 2.30 | 1.16E-21 | Conserved |
| SAUSA300_0858 | *-* | 1.88 | 2.72E-20 | Degradation of RNA |
| SAUSA300_1968 | *-* | 2.15 | 6.28E-11 | DNA interactions |
| SAUSA300_0804 | *-* | 3.11 | 8.73E-31 | DNA interactions |
| SAUSA300_0805 | *-* | 3.47 | 3.20E-54 | DNA interactions |
| SAUSA300_1238 | *-* | 1.13 | 2.19E-08 | DNA replication, recombination, and repair |
| SAUSA300_1958 | *-* | 2.19 | 5.13E-14 | DNA replication, recombination, and repair |
| SAUSA300_0777 | *-* | 1.11 | 4.49E-03 | DNA replication, recombination, and repair |
| SAUSA300_2639 | *-* | 1.16 | 8.25E-08 | DNA replication, recombination, and repair |
| SAUSA300_1957 | *-* | 1.15 | 1.87E-02 | DNA replication, recombination, and repair |
| SAUSA300_0809 | *-* | 2.75 | 2.08E-39 | DNA replication, recombination, and repair |
| SAUSA300_1960 | *-* | 3.04 | 1.48E-67 | DNA replication, recombination, and repair |
| SAUSA300_0270 | *lytM* | 1.22 | 2.06E-10 | DNA-dependent RNA polymerase |
| SAUSA300_1967 | *-* | 2.10 | 9.15E-31 | Enzymes of unknown specificity |
| SAUSA300_0732 | *-* | 1.07 | 1.48E-07 | General |
| SAUSA300_0788 | *-* | 1.15 | 7.81E-13 | Heme, porphyrin, and cobalamin |
| SAUSA300_0542 | *-* | 1.22 | 1.48E-07 | Nucleotide and nucleoside interconversions |
| SAUSA300_0812 | *-* | 1.69 | 2.50E-35 | Prophage functions |
| SAUSA300_1944 | *-* | 1.36 | 2.23E-06 | Prophage functions; DNA interactions |
| SAUSA300_1712 | *ribH* | 2.95 | 1.73E-127 | Riboflavin, FMN, and FAD |
| SAUSA300_1713 | *ribBA* | 3.21 | 3.46E-170 | Riboflavin, FMN, and FAD |
| SAUSA300_1715 | *ribD* | 3.36 | 5.21E-144 | Riboflavin, FMN, and FAD |
| SAUSA300_1714 | *ribE* | 3.47 | 1.26E-186 | Riboflavin, FMN, and FAD |
| SAUSA300_0591 | *-* | 1.25 | 9.77E-14 | Ribosomal proteins: synthesis and modification |
| SAUSA300_1117 | *rpmB* | 1.77 | 7.32E-16 | Ribosomal proteins: synthesis and modification |
| SAUSA300_1535 | *rpsU* | 1.80 | 1.47E-19 | Ribosomal proteins: synthesis and modification |
| SAUSA300_1260 | *-* | 1.49 | 1.10E-24 | Serine family |
| SAUSA300_0438 | *-* | 1.01 | 2.22E-11 | Sporulation and germination |
| SAUSA300_0651 | *-* | 1.45 | 4.49E-11 | Sporulation and germination |
| SAUSA300_2253 | *ssaA* | 2.24 | 2.73E-45 | Surface structures |
| SAUSA300_0373 | *-* | 1.37 | 6.38E-09 | tRNA and rRNA base modification |

|  | **Downregulated genes** | | | |
| --- | --- | --- | --- | --- |
| **Locus tag** | **Gene** | **log2FoldChange** | **FDR** | **TIGRFAM Sub Role** |
| SAUSA300_1331 | *ald* | -1.65 | 6.42E-13 | Amino acids and amines |
| SAUSA300_2278 | *hutU* | -1.22 | 1.97E-17 | Amino acids and amines |
| SAUSA300_2192 | *rplE* | -1.22 | 8.63E-27 | Amino acids and amines |
| SAUSA300_2277 | *hutI* | -1.13 | 2.14E-13 | Amino acids and amines |
| SAUSA300_1417 | *-* | -1.04 | 4.12E-02 | Amino acids and amines |
| SAUSA300_1329 | *-* | -1.61 | 3.98E-11 | Amino acids, peptides and amines |
| SAUSA300_2342 | *narH* | -2.80 | 1.60E-43 | Anaerobic |
| SAUSA300_2343 | *-* | -2.32 | 3.97E-42 | Anaerobic |
| SAUSA300_2333 | *narK* | -1.79 | 2.57E-38 | Anions |
| SAUSA300_2476 | *ptsG* | -1.66 | 2.69E-25 | Carbohydrates, organic alcohols, and acids |
| SAUSA300_2313 | *-* | -1.46 | 1.34E-17 | Carbohydrates, organic alcohols, and acids |
| SAUSA300_1874 | *-* | -1.87 | 9.11E-46 | Cations and iron carrying compounds |
| SAUSA300_0666 | *-* | -1.21 | 1.27E-21 | Conserved |
| SAUSA300_2191 | *rpsN* | -1.18 | 1.16E-20 | Conserved |
| SAUSA300_2161 | *hysA* | -1.07 | 1.09E-16 | Conserved |
| SAUSA300_1755 | *splD* | -1.40 | 1.00E-13 | Degradation of proteins, peptides, and glycopeptides |
| SAUSA300_1753 | *splF* | -1.30 | 4.50E-10 | Degradation of proteins, peptides, and glycopeptides |
| SAUSA300_1754 | *splE* | -1.25 | 2.90E-10 | Degradation of proteins, peptides, and glycopeptides |
| SAUSA300_1757 | *splB* | -1.35 | 6.16E-13 | Degradation of proteins, peptides, and glycopeptides |
| SAUSA300_1756 | *splC* | -1.27 | 4.56E-12 | Degradation of proteins, peptides, and glycopeptides |
| SAUSA300_1758 | *splA* | -1.26 | 4.75E-11 | Degradation of proteins, peptides, and glycopeptides |
| SAUSA300_2097 | *-* | -1.03 | 7.65E-24 | Enzymes of unknown specificity |
| SAUSA300_0220 | *pflB* | -1.75 | 9.00E-19 | Fermentation |
| SAUSA300_0594 | *adh* | -1.67 | 2.15E-18 | Fermentation |
| SAUSA300_2344 | *-* | -1.34 | 1.45E-15 | Heme, porphyrin, and cobalamin |
| SAUSA300_2346 | *nirB* | -1.86 | 4.70E-20 | Nitrogen metabolism |
| SAUSA300_2345 | *nirD* | -1.56 | 4.98E-08 | Nitrogen metabolism |
| SAUSA300_1092 | *pyrP* | -2.01 | 4.97E-07 | Nucleosides, purines and pyrimidines |
| SAUSA300_0815 | *ear* | -1.18 | 9.93E-10 | Prophage functions |
| SAUSA300_0283 | *-* | -1.04 | 2.67E-18 | Protein and peptide secretion and trafficking |
| SAUSA300_2341 | *narJ* | -2.66 | 6.09E-20 | Protein folding and stabilization |
| SAUSA300_0877 | *clpB* | -1.15 | 1.40E-19 | Protein folding and stabilization |
| SAUSA300_0221 | *pflA* | -2.08 | 3.61E-25 | Protein modification and repair |
| SAUSA300_0017 | *purA* | -1.04 | 7.73E-10 | Purine ribonucleotide biosynthesis |
| SAUSA300_1096 | *carB* | -2.85 | 2.53E-65 | Pyrimidine ribonucleotide biosynthesis |
| SAUSA300_1094 | *pyrC* | -2.84 | 4.46E-53 | Pyrimidine ribonucleotide biosynthesis |
| SAUSA300_1095 | *carA* | -2.82 | 9.17E-59 | Pyrimidine ribonucleotide biosynthesis |
| SAUSA300_1093 | *pyrB* | -2.71 | 6.36E-36 | Pyrimidine ribonucleotide biosynthesis |
| SAUSA300_1097 | *pyrF* | -2.61 | 1.82E-51 | Pyrimidine ribonucleotide biosynthesis |
| SAUSA300_1098 | *pyrE* | -2.49 | 2.23E-56 | Pyrimidine ribonucleotide biosynthesis |
| SAUSA300_2081 | *pyrG* | -1.09 | 2.99E-17 | Pyrimidine ribonucleotide biosynthesis |
| SAUSA300_1330 | *ilvA* | -2.08 | 1.46E-16 | Pyruvate family |
| SAUSA300_2193 | *rplX* | -1.24 | 3.19E-21 | Ribosomal proteins: synthesis and modification |
| SAUSA300_2188 | *rplR* | -1.22 | 1.35E-21 | Ribosomal proteins: synthesis and modification |
| SAUSA300_2186 | *rpmD* | -1.16 | 9.49E-16 | Ribosomal proteins: synthesis and modification |
| SAUSA300_2197 | *rplP* | -1.14 | 1.64E-14 | Ribosomal proteins: synthesis and modification |
| SAUSA300_2187 | *rpsE* | -1.13 | 4.83E-12 | Ribosomal proteins: synthesis and modification |
| SAUSA300_2195 | *rpsQ* | -1.13 | 4.38E-16 | Ribosomal proteins: synthesis and modification |
| SAUSA300_2199 | *rplV* | -1.08 | 7.99E-23 | Ribosomal proteins: synthesis and modification |
| SAUSA300_2196 | *rpmC* | -1.08 | 2.21E-09 | Ribosomal proteins: synthesis and modification |
| SAUSA300_2198 | *rpsC* | -1.07 | 6.08E-13 | Ribosomal proteins: synthesis and modification |
| SAUSA300_2194 | *rplN* | -1.06 | 9.56E-10 | Ribosomal proteins: synthesis and modification |
| SAUSA300_2189 | *rplF* | -1.05 | 3.04E-10 | Ribosomal proteins: synthesis and modification |
| SAUSA300_2200 | *rpsS* | -1.02 | 7.99E-14 | Ribosomal proteins: synthesis and modification |
| SAUSA300_1890 | *-* | -1.40 | 3.38E-18 | RNA processing |
| SAUSA300_0311 | - | -1.06 | 5.71E-03 | Sugars |
| SAUSA300_0212 | - | -1.02 | 4.02E-11 | Sugars |
| SAUSA300_1058 | - | -1.59 | 3.20E-24 | Toxin production and resistance |
| SAUSA300_1974 | - | -1.37 | 1.41E-13 | Toxin production and resistance |
| SAUSA300_1381 | *lukF-PV* | -1.23 | 2.79E-19 | Toxin production and resistance |
| SAUSA300_1382 | *lukS-PV* | -1.20 | 2.81E-18 | Toxin production and resistance |
| SAUSA300_1975 | - | -1.18 | 6.62E-09 | Toxin production and resistance |
| SAUSA300_2399 | - | -1.01 | 1.35E-09 | Toxin production and resistance |

**Supplementary Figure 1.** Loss of function in other *pyr* genes from NTML does not affect bacterial growth in tryptic soy broth medium. These results represent the mean ± SD of three independent experiments (four replicates for each strain).

**Supplementary Figure 2.**  Loss of function in other *pyr* genes from NTML does not affect the H_2_O_2_ sensitivity. Results represent the mean ± SD of two independent experiments (four replicates for each H_2_O_2_ concentration). A nonlinear regression model was made to estimate the IC_50_.
